# Integrating soil health and manure practices to advance sustainability for US dairy farms

**DOI:** 10.1002/jeq2.70202

**Published:** 2026-06-13

**Authors:** Mara L. Cloutier, Sam Zipper, Daniel Liptzin, E. Carol Adair, Brent W. Auvermann, Jourdan M. Bell, Bert Bock, Carolina B. Brandani, Dennis Busch, Nicholas E. Clark, Karl J. Czymmek, Joshua W. Faulkner, Adam C. von Haden, William R. Horwath, Randall D. Jackson, Quirine M. Ketterings, Sat Darshan S. Khalsa, Deanne Meyer, Gregg R. Sanford, James Wallace, Reza K. Afshar, Cristine L. S. Morgan

**Affiliations:** ^1^ Soil Health Institute Morrisville North Carolina USA; ^2^ Kansas Geological Survey University of Kansas Lawrence Kansas USA; ^3^ Department of Geology University of Kansas Lawrence Kansas USA; ^4^ Rubenstein School of Environment and Natural Resources University of Vermont Burlington Vermont USA; ^5^ Texas A&M AgriLife Research Amarillo Texas USA; ^6^ BR Bock Consulting, Inc. Athens Georgia USA; ^7^ School of Agriculture University of Wisconsin‐Platteville Elk Grove Wisconsin USA; ^8^ University of California Cooperative Extension Hanford California USA; ^9^ PRO‐DAIRY Program Cornell University Ithaca New York USA; ^10^ Extension Center for Sustainable Agriculture University of Vermont Burlington Vermont USA; ^11^ Department of Soil & Environmental Sciences University of Wisconsin‐Madison Madison Wisconsin USA; ^12^ Department of Land, Air and Water Resources University of California Davis California USA; ^13^ Department of Plant & Agroecosystem Sciences University of Wisconsin‐Madison Madison Wisconsin USA; ^14^ Department of Animal Science Cornell University Ithaca New York USA; ^15^ Department of Plant Sciences, College of Agricultural and Environmental Sciences University of California Davis California USA; ^16^ Department of Animal Science University of California Davis California USA; ^17^ Dairy Research Institute Rosemont Illinois USA

## Abstract

The US dairy industry has committed to advancing environmental sustainability by reducing greenhouse gas (GHG) emissions, enhancing water use efficiency, and improving water quality. Feed production accounts for approximately 12% of GHG emissions and 99% of consumptive water use from dairy operations, making it a focus area for potential resource use and overall efficiency improvements. However, few studies report changes to GHG emissions or water quantity and quality outcomes from adopting soil health management systems and use of novel manure products for dairy feed production. The Dairy Soil and Water Regeneration (DSWR) project is exploring whether soil health management systems and novel manure products can help advance environmental sustainability outcomes across major dairy‐producing regions in the United States. Through a suite of coordinated studies, including regional soil benchmarking and large‐plot‐ to field‐scale experiments, DSWR is evaluating the performance and scalability of reduced tillage, cover crops, and novel manure products applied to row crop feed production systems. By integrating high‐resolution data on soil, water, GHG emissions, and crop production, this project is generating actionable insights to support decision‐making for farmers, farm managers, dairy cooperatives, retailers, and consumer packaged goods companies. We introduce the project by summarizing its purpose, the conceptual framework guiding its design and implementation, and its approaches to hypothesis testing about soil health, hydrology, and yield responses.

## Plain Language Summary

The US dairy industry is working to reduce greenhouse gas emissions, improve water use efficiency, and protect water quality. Producing feed for dairy cows accounts for about 12% of total greenhouse gas emissions from dairy farms and virtually all of the dairy industry's water use, while most of the potential for water quality impairment on a dairy farm is also associated with growing feed. Thus, improving feed production offers a major opportunity to advance environmental sustainability. The Dairy Soil and Water Regeneration project is testing whether soil health management practices and new manure‐based products can reduce emissions and improve soil and water outcomes in key dairy regions. By collecting detailed data on soil, water, greenhouse gases, and crop yields, the project is generating practical insights to guide farmers, cooperatives, and food companies toward practices that improve both environmental performance and dairy farm sustainability.

**Abbreviations**
CNV: conventional
DSWR: Dairy Soil and Water Regeneration
GHG: greenhouse gas
SHM: soil health management

## 1 INTRODUCTION

Dairy milk is a major agricultural product in the US accounting for approximately 15% of global annual milk production (USDA‐FAS, 2024/2025). Dairy operations in the United States are broadly categorized as confinement‐ or grazing‐based, a distinction that has important implications for feed production, nutrient management, and environmental outcomes. Most dairy cows are currently in confinement‐based systems, where feed is mechanically harvested, mixed, and delivered to cows in a barn or open‐lot. Given the dominant role of confinement‐based dairies in US milk production currently (Rotz et al., 2021), improving feed production practices within these systems is an important part of achieving the US dairy industry environmental stewardship goals (2050 ES goals): (1) achieving greenhouse gas (GHG) neutrality, (2) improving water use efficiency, and (3) improving water quality by 2050 (Innovation Center for U.S. Dairy, 2023).

However, the existing literature evaluating how using single or multiple practices under real‐world farm conditions in dairy forage systems, such as cover crops, reduced tillage, and novel manure products, is limited to a work in a handful of states, insufficient to support practice change across the United States (Cloutier et al., 2025). Current research lacks evaluation of combined management practices with measured, comprehensive environmental outcomes across a sufficient long (5+ year) study duration to observe slowly changing variables. While the environmental impacts of a dairy farm can occur in many parts of the operation, feed production is an important component of total GHG emissions (Rotz et al., 2021), water use (Mekonnen et al., 2019), and nitrate leaching (van der Schans, 2009). Further research is necessary to assess the potential contributions of these practices to the 2050 ES goals, their scalability, and economic feasibility to support the long‐term sustainability of dairy operations.

The Dairy Soil and Water Regeneration (DSWR) project was developed to evaluate how soil health management (SHM) practices and novel manure products affect soil health, GHG emissions, water quality and quantity, forage yield, and forage quality within confinement‐based dairy feed production systems. Here, soil health is defined as the continued capacity of soil to function as a vital living ecosystem that sustains plants, animals, and humans (USDA‐NRCS, 2025). Accordingly, soil health management practices are those that apply one or more of the soil health principles of minimizing disturbance, maximizing soil cover, maintaining living roots, and maximizing biodiversity (USDA‐NRCS, 2025). The environmental benefits of SHM practices depend on how comprehensively and effectively the soil health principles are applied and local environmental conditions (Augarten et al., 2023; Hsiao et al., 2026; Summers et al., 2025).

Feed production is central to achieving the 2050 ES goals, as soil and manure management directly influence GHG emissions, water quantity, and water quality outcomes. Dairy forage production and manure management account for approximately 12.1% and 24.5% of total farm‐level GHG emissions, respectively (Pelton et al., 2025). Feed production also strongly shapes water quantity through its effects on water use, infiltration, and soil water storage, and feed production is estimated to constitute 99% of the total consumptive water use of dairy production (Mekonnen et al., 2019). However, water use efficiency has been rarely evaluated under commercial‐scale dairy operations, limiting understanding of real‐world performance (Cloutier et al., 2025). SHM practices, such as cover crops, reduced tillage and diversified crop rotations, have been shown to improve water infiltration and retention across cropping systems globally (Basche & Delonge, 2019; Hatfield et al., 2001; Yan & Arthur, 2025). In addition, feed production is a major driver of water quality outcomes, as nutrient losses from cropland contribute to nitrogen (N) and phosphorus (P) pollution to surface and groundwater systems (Lane et al., 2020; Motew et al., 2017). A recent modeling study estimated that adoption of soil health, manure, and fertilizer management practices has reduced these nutrient losses by 27%–51% over the past 50 years (Rotz et al., 2024). However, nutrient losses persist due to system‐level drivers, including manure nutrient surpluses, dairy consolidation, and soil management, which elevate risks of accumulation, runoff, and eutrophication (Dell et al., 2022; MacDonald et al., 2007; van Meter et al., 2018). Together, these findings highlight the need for integrated feed production strategies that reduce GHG emissions, enhance water use efficiency, and improve water quality across dairy systems in the United States.

## 2 THE DSWR PROJECT

**FIGURE 1:**
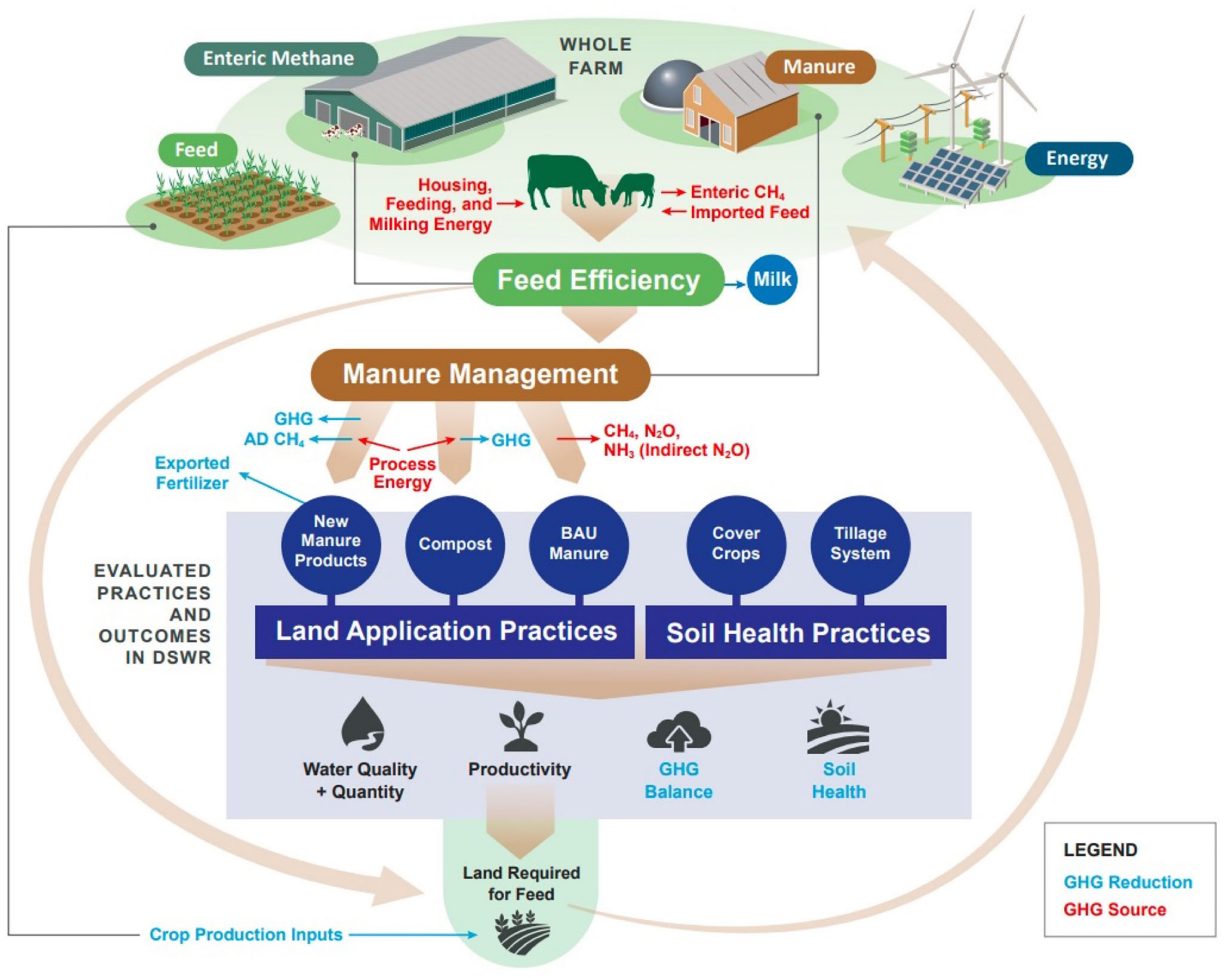
Whole farm conceptual model showing integration of the Dairy Soil and Water Regeneration (DSWR) project. DSWR focuses on feed production and alternative manure products in contrast to conventional manure products and practices. In the diagram, key greenhouse gas (GHG) sources are shown, and with hypothesized reductions in GHG emissions associated with advanced soil health management, new manure products, and compost shown in light blue text and hypothesized increases in GHG emissions shown as red text. AD, anaerobically digested manure.

The DSWR project was launched in 2021 to advance understanding of how to achieve the 2050 ES goals of the US dairy industry in crop production systems. It is a multi‐institutional and multistate effort designed to examine how SHM systems, hydrologic conditions, and application of novel manure products interact to influence key agronomic and environmental outcomes. While the project focuses on the feed production component of confinement‐based dairy farms, it connects more broadly to the 2050 ES goals. For example, improving crop productivity through SHM could reduce the total land needed for feed, improving feed efficiency and lowering methane emission intensity (Figure 1). Ultimately, DSWR is designed to generate the scientific evidence needed to guide adoption of improved practices across the industry and to support development of new carbon and water quality markets.

**FIGURE 2:**
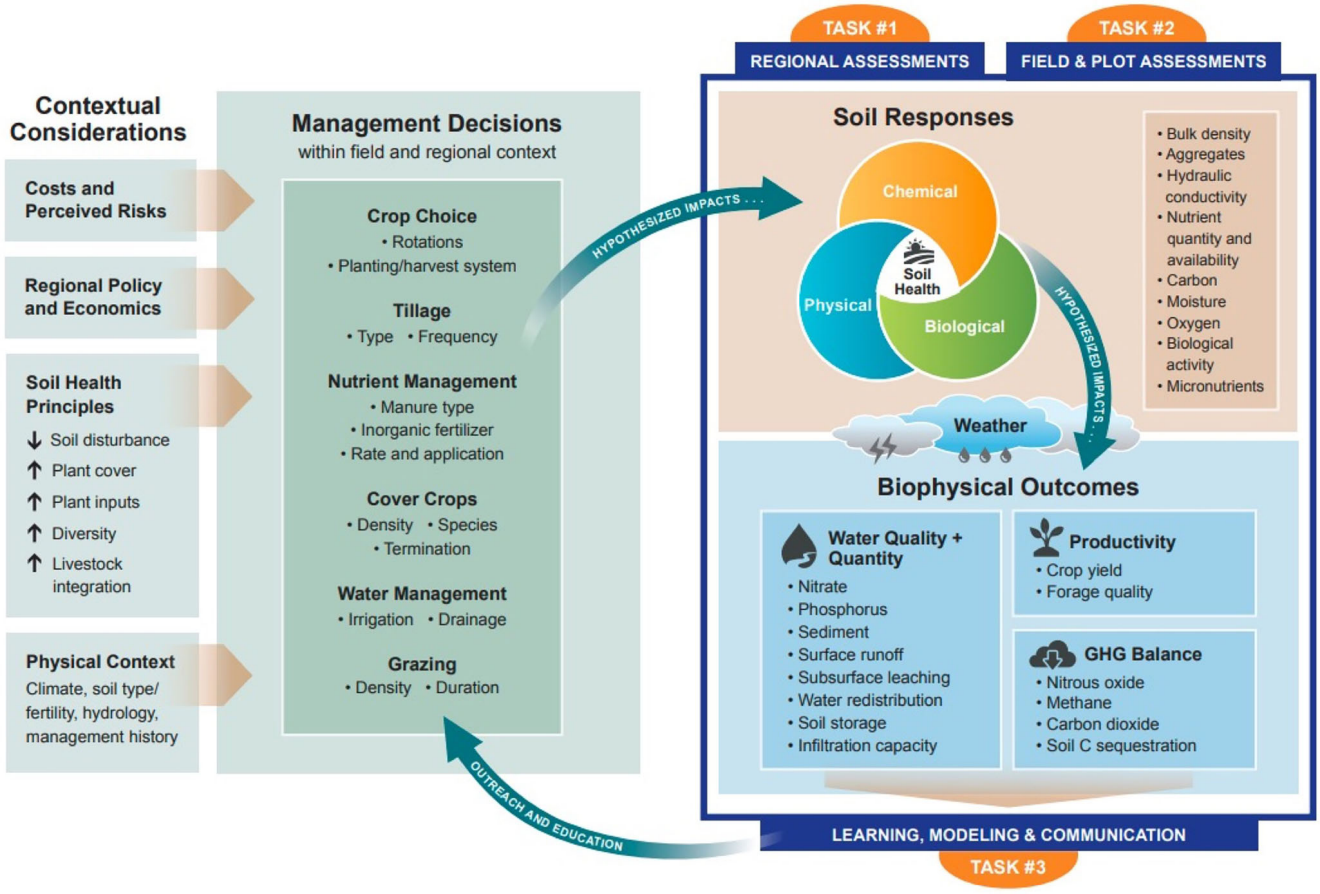
Conceptual model showing the Dairy Soil and Water Regeneration (DSWR) project design, in which field and regional contextual considerations shape management decisions. The impacts of these management decisions are then evaluated and shared through DSWR's three tasks. The contextual considerations helped to shape the region‐specific management decisions to (1) target for Task 1 sampling and (2) implement and monitor in Task 2. Measured parameters from Task 1 and Task 2 are included in the “Soil Responses” box and the agronomic and environmental outcomes measured are included in the “Biophysical Outcomes” box.

To accomplish this, the DSWR project evaluates how adoption of selected SHM practices affects soil health and associated agroecosystem services across the major dairy regions in the United States. The studied practices include reduced/no‐tillage, cover crops, and novel manure products. The geography of the DSWR project spans four major dairy‐producing regions in the United States: the Northeast, the Lake States, the Mountain Region, and the Pacific Region, which collectively account for nearly 80% of national milk production. These regions were selected to assess how variation in climate, soil types, and logistical and management constraints influence SHM and novel manure product performance. By working across a range of conditions, DSWR seeks to identify region‐specific management practices that support the 2050 ES goals while still maintaining crop production. Specifically, DSWR measures indicators of crop production, soil organic carbon, soil P and N availability, water quality, soil health metrics, and water use efficiency (Figure 2). These measurements enable integrated assessment of SHM systems in terms of both environmental and agronomic outcomes.

The SHM practices studied in DSWR are tailored to regional forage production systems and are assessed through three research components (referred to as “Tasks”), each with a specific objective:

1. Task 1: Regional measurements to benchmark soil health and soil carbon stocks on geographically dispersed dairies across the United States.
2. Task 2: Field‐ and plot‐scale experiments to quantify GHG emissions, nutrient management, soil carbon storage, and water quality/quantity outcomes under novel SHM systems and novel manure products.
3. Task 3: Biophysical modeling and communication to socialize the learnings from Tasks 1 and 2 to the dairy community and improve ecosystem and carbon model development.

The hypotheses informing the experimental designs (outlined in the next section) are illustrated in Figure 2. Management decisions at the farm‐ or field‐level are shaped by broader contextual factors, including policy, economics, soil health principles, and physical conditions such as climate and hydrology. These management decisions impact soil health and, in concert with external factors such as weather, influence field‐scale biophysical outcomes. Hypothesized directional relationships are not specified in this conceptual model because we expect the directions and strengths of relationships among soil responses and biophysical outcomes to vary across regions. Knowledge generated from Tasks 1 and 2 will be linked back to decision‐making through outreach, education, and modeling in Task 3.

## 3 DSWR RESEARCH COMPONENTS

### 3.1 Regional assessment

**FIGURE 3:**
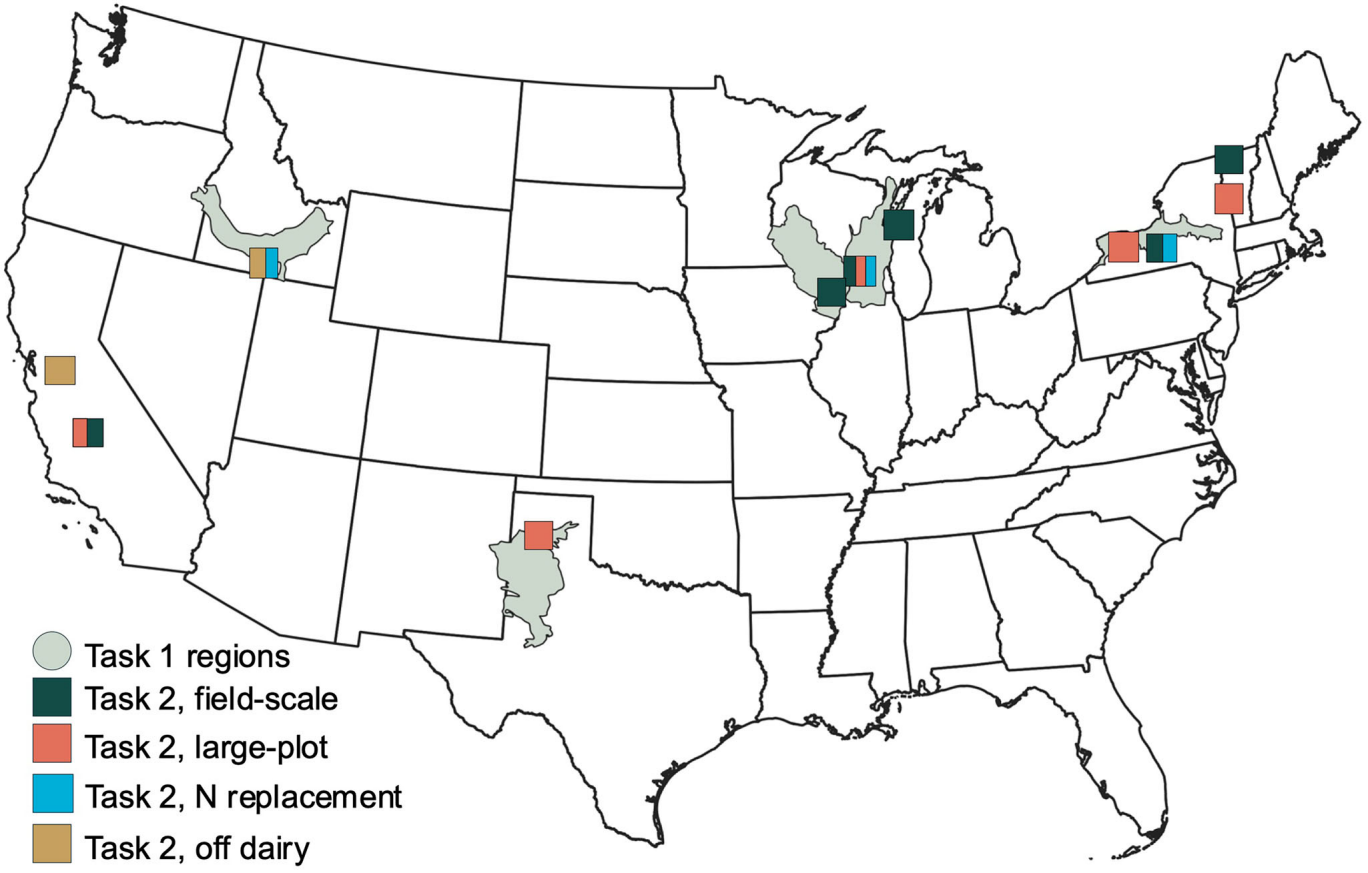
Sampling regions for Task 1 and locations of the experimental sites associated with Task 2. The specific experiments at each site are indicated in Table 1.

Task 1 was aimed at quantifying the baseline state of soil health and carbon stocks in dairy feed production fields in Major Land Resource Areas of five regions: the Snake River Plains in Idaho, the Ontario‐Erie Plain and Finger Lakes in New York, the Southern Part of the Southern High Plains in Texas and New Mexico, and both the Upper Mississippi River Bedrock Controlled Uplands and Valleys and the Eastern Wisconsin, Northern Illinois, and Upper Michigan Drift Plain in Wisconsin (Figure 3). Selected fields needed to be within the identified major land resource area, on a soil type that widely occurs in the region, have a history of manure amendments, and be used to grow forage for dairy cows. Fields were selected based on volunteer farmer enrollment and, where possible, fields that represented contrasting SHM practices. Based on the site selections, we tested the following hypotheses:

1. Regions will differ in soil health indicator values based on differences in climate and soil texture.
2. Fields managed with soil health practices will have greater soil health indicator values after controlling for climate and soil texture within each region.

To test these hypotheses, we collected samples from 271 sampling locations across 211 fields. At each field a set of soil samples (composite and intact) was collected from a circle with a 5 m radius. The Soil Health Institute's recommended essential measurements for soil health assessments (soil organic carbon, carbon mineralization potential, available water holding capacity, and wet aggregate stability) were collected following the Soil Health Institute's Standard Operating Procedure for Soil Health Sampling (Bagnall et al., 2023; Soil Health Institute, 2024). Water holding capacity was measured using intact cores (7.4 cm diameter and 0‐ to 15‐cm depth). Additional measurements from the 0‐ to 15‐cm composite samples included inorganic carbon, total nitrogen, pH, soluble salts, and texture to aid in our interpretation of the soil health indicators (Looker et al., 2025). Additional details about this experiment and initial results can be found in Hsiao et al. (2026).

### 3.2 Field‐scale and large‐plot studies

Task 2 was designed to assess how changes in soil health and manure management practices for dairy feed production affect environmental and agronomic outcomes, and how these impacts vary under different hydrologic conditions. Experiments within Task 2 include field‐scale and large‐plot studies, some of which also evaluate water quality or quantity, the N replacement value of the novel manure products, or the use of novel manure products on off‐dairy fields (Table 1; Figure 3). Field‐scale studies are on farm fields ranging from 0.30 to 22.76 ha, while large‐plot studies have replicated plots with an average size of 0.50 ha. Each of these is briefly detailed below.

**TABLE 1:** Site characteristics for experiments within Task 2.

Site	State	Experiment	Water management	Soil texture	MAT (°C)	MAP (mm)
CD	California	Off‐dairy, on‐farm	Micro sprinklers	Fine sandy loam, 0%–2% slope	17.2	372
CF	California	Field‐scale, on‐farm	Flood irrigation	Fine sandy loam, 0%–2% slope	18.1	209
CP	California	Large‐plot, on‐farm	Flood irrigation	Fine sandy loam, 0%–2% slope	18.1	209
IK	Idaho	Off‐dairy, research site	Sprinkler irrigation	Silt loam, 0%–2% slope	8.6	408
IN	Idaho	N‐replacement, research site	Sprinkler irrigation	Silt loam, 0%–2% slope	8.6	408
NO	New York	Field‐scale, on‐farm	Rainfall only	Silt loam, 0%–3% and 3%–8% slopes	8.4	1052
NP	New York	Large‐plot, on‐farm	Rainfall only	Loam, 0%–3% slope	8.6	999
NR	New York	N‐replacement, on‐farm	Rainfall only	Silt loam, 0%–3% and 3%–8% slopes and loam, 3%–8% slope	8.8	981
TA	Texas	Large‐plot, research site	Center pivot	Clay loam, 1%–3% slope and silty clay loam, 0%–1% slope	13.9	529
VA	Vermont	Large‐plot, on‐farm	Rainfall only	Clay, 0%–3% slope	7.8	978
VB	Vermont	Field‐scale, on‐farm	Rainfall only	Silty clay, 0%–3% slope and clay, 2%–6% and 12%–25% slopes	8.3	1002
WA	Wisconsin	Large‐plot, research site	Rainfall only	Silt loam, 0%–2%, 2%–6%, and 6%–12% slopes	7.8	993
WF	Wisconsin	Field‐scale, research site	Rainfall only	Silt loam, 0%–2%, 0%–4%, and 2%–6% slopes	7.8	998
WK	Wisconsin	Field‐scale, on‐farm	Rainfall only	Silt loam, 0%–3% and 2%–6% slopes	6.9	853
WN	Wisconsin	N‐replacement, research site	Rainfall only	Silt loam, 0%–2% slope	7.9	1001
WP	Wisconsin	Field‐scale, research site	Rainfall only	Silt loam, 2%–6% and 6%–12% slopes	8.1	1032

Data collected at the field‐scale, large‐plot, and off‐dairy experimental sites include yearly soil sampling to measure SHI's essential soil health indicators (0–15 cm) and soil carbon stocks (0–30 cm); surface GHG emissions to include carbon dioxide (CO_2_), methane (CH_4_), and nitrous oxide (N_2_O); soil oxygen and moisture measurements (hourly); deep cores every other year for soil organic carbon (0–1 m or limiting layer), total nitrogen (N), and nitrate (NO_3_^−^); intact cores for available water holding capacity every other year; and saturated hydraulic conductivity at the soil surface every other year. Harvested forages are sampled for stalk nitrate (corn and sorghum) and forage quality. Forage yield data are collected using yield monitor systems where possible; otherwise biomass samples are collected to quantify forages and cover crops. Manure samples are collected at the time of application to quantify nutrient concentrations, moisture content, and organic matter. A full list of measurements and methods are presented in Table 2.

**TABLE 2:** Measured soil and agronomic properties for the Dairy Soil and Water Regeneration project along with the analytical method where appropriate.

Sample type	Sites	Measurements	Depth (cm)	Timing	Method	Reference
Soil health	All large‐plot and field‐scale sites	Soil organic carbon	0–15; 15–30	Once per year, 4 weeks after spring planting	Dry combustion, corrected for inorganic C, if present, using a modified pressure calcimeter method	(Nelson & Sommers, 1996; Sherrod et al., 2002)
		Total nitrogen			Dry combustion	(Nelson & Sommers, 1996)
		Bulk density			Core method, sieving for rocks if estimated at >2%	(Blake & Hartge, 1986)
		Aggregate stability	0–6		Wet aggregate stability, image‐based analysis of soil slaking	(Fajardo et al., 2016)
		Carbon mineralization potential	0–15		2‐day incubation followed by CO_2_‐C evolved at 50% water filled pore space	(Zibilske, 1994)
		pH			1:2 soil:water	(Thomas, 1996)
		Electrical conductivity			1:2 soil:water	(Rhoades, 1996)
		P, K, Ca, Mg, S			Mehlich‐3 extractant	(Olsen & Sommers, 1982; Knudsen et al., 1982)
		Available water holding capacity		Every other year, 4 weeks after spring planting	Tension table at −33 kPa on intact cores and −1500 kPa on pressure plates with repacked cores	(Topp et al., 1993; Reynolds & Topp, 2008)
		Saturated hydraulic conductivity	Surface	Every other year, either fall or spring	Two‐ponding head method	(Reynolds & Elrick, 1990)
Deep soil cores	All large‐plot and field‐scale sites	Soil organic carbon	0–15; 15–30; 30–45; 45–60; 60–100	Every other year, either fall or spring	Dry combustion, corrected for inorganic C, if present, using a modified pressure calcimeter method	(Nelson & Sommers, 1996; Sherrod et al., 2002)
		Bulk density			Core method, sieving for rocks if estimated at >2%	(Blake & Hartge, 1986)
		Nitrate			Cadmium reduction method, potassium chloride extract	(Carson, 1980)
		Total nitrogen			Dry combustion	(Nelson & Sommers, 1996)
		Soil texture		First sample only	Hydrometer method	(Gee & Bauder, 1986)
GHG measurements	All large‐plot and field‐scale sites except WK and VA	Soil CH_4_, CO_2_, N_2_O fluxes	Surface	Once per week during growing season to once per month. More frequent after manure addition and rainfall or irrigation events.	LI‐COR 7810/7820 and Smartchamber, when rows are present, one in‐row and one out‐of‐row	‐
		Soil temperature	5		Omega thermocouple	
		Soil moisture			Stevens hydroprobe	
	CD, NO, NP, VB, WA, WF, WN	Extractable nitrate	Varies by site		Varies by site	‐
		Extractable ammonium	Varies by site			‐
Soil microclimate	TA	Soil moisture	10‐ to 230‐ in 20 cm‐ increments	Once per week	Neutron probe	‐
	All large‐plot and field‐scale sites	Soil moisture and temperature	10	Every 15 min	Meter TEROS 11	‐
		Soil oxygen			Apogee SO411	‐
Weather	All large‐plot and field‐scale sites	Precipitation	‐		Meter ECRN‐100	‐
		Air temperature, relative humidity, barometric pressure	‐		Meter ATMOS	‐
Forage productivity	All sites	Yield	‐	During harvest	Hand harvest, truck weights, yield monitor	‐
Forage quality	All sites except CD and VA	Dry matter	‐	During harvest	Grab samples from harvester	‐
		Ash	‐			(Thiex et al., 2012)
		Crude protein	‐			(Hall et al., 1999)
		Soluble protein	‐			(Krishnamoorthy et al., 1982)
		Ca, P, Mg, K, Na, Fe, Mn, Zn, and Cu	‐			(AOAC International Publications, 2023)
		pH	‐			
		Acid detergent fiber	‐			(AOAC International Publications, 2000)
		Neutral detergent fiber	‐			(van Soest et al., 1991)
Plant nutrition	All sites except CD	Corn‐stalk nitrate	‐	Before corn or sorghum harvest	Hand harvest	‐
	CD	Almond leaf tissue nutrients: N, P, K, S, B, Ca, Mg, Zn, Mn, Fe, Cu, Na	‐	Once per harvest	Dry combustion for N, nitric acid digestions for others	‐
Manure	All sites	Dry matter, ash, pH, total nitrogen, nitrate, ammonium, P, K, S	‐	During application	Varies by site	‐

*Manure products* tested in the field‐scale, large‐plot, and the N replacement experiments were selected based on regional needs and the potential of alternative manure fertilizers to provide additional ecosystem benefits (Tables 3 and 4). Processing liquid dairy manure into products that allow dairy farmers to better control N and P balances on their fields should provide measurable benefits to ecosystem services (Gamble & Alexander, 2025), as tested in this project. The novel manure products tested include two forms of flocculated solids, an evaporative solid, and a semi‐composted dairy manure. Manure properties for two experimental sites in New York are presented in Table 5 as examples of typical values.

**TABLE 3:** Dairy feed production practices implemented for conventional (CNV) and soil health management (SHM) systems for field‐scale experiments (Task 2). These studies were designed to take a systems‐level approach to assessing forage management.

Sites	Treatments	Tillage	Manure types tested	Application methods and timing	Rotation
CF, California	CNV	Full width	Liquid	Flood irrigated, blended in multiple in‐season irrigations	Sorghum–triticale double crop
	SHM	Reduced till	Windrow‐dried solids	Broadcast, preplant spring and after fall harvest	Sorghum–triticale double crop
NO, New York	CNV	Full width	Liquid	Broadcast incorporated, spring and after fall harvest split applications	Corn silage–wheat cover
	SHM	No‐till	Semi‐compost	Broadcast, spring	Corn silage–rye cover
WK, Wisconsin	CNV	Full width	Liquid	Broadcast, after fall harvest	Corn silage
	SHM	Strip till	Compost, evaporative, and flocculated solids	Broadcast, spring	Corn silage–oats/rye cover
WF, Wisconsin	CNV	Full width	Liquid	Broadcast incorporated, after fall harvest	Corn silage
	SHM	Strip till	Compost/evaporative solids	Broadcast, after fall harvest	Corn silage–rye cover
WP, Wisconsin	CNV	Full width	Liquid	Injected, after fall harvest	Corn silage
	SHM‐1	No‐till	Evaporative and flocculated solids	Broadcast, spring	Corn silage–rye cover
VB, Vermont	CNV	Full width	Liquid	Broadcast incorporated, after fall harvest	Corn silage
	SHM‐1	No‐till + strip till	Liquid	Injected, after fall harvest	Corn silage–wheat cover
	SHM‐2	No‐till + strip till	Flocculated liquid	Broadcast, after fall harvest	Corn silage–wheat cover

**TABLE 4:** Dairy feed production practices implemented for conventional (CNV) and soil health management (SHM) treatments in the large‐plot studies (Task 2). These are replicated experiments designed to assess combinations of soil health practices including no‐till or strip tillage, cover crops, and novel manure products.

Sites	Treatments	Tillage	Manure types tested	Manure application methods and timing	Rotation
CP, California	CNV‐1	Full width	Liquid	Flood irrigated, blended in multiple in‐season irrigations	Sorghum–triticale double crop
	CNV‐2	Full width	Untreated manure solids	Broadcast incorporated, preplant spring and fall	Sorghum–triticale double crop
	CNV‐3	Full width	Windrow‐dried solids	Broadcast incorporated, preplant spring and fall	Sorghum–triticale double crop
	SHM‐1	Strip + no‐till	Windrow‐dried solids	Broadcast solids, preplant spring and fall	Sorghum–triticale double crop
	SHM‐2	Strip + no‐till	None	None	Sorghum–triticale double crop
	SHM no N control	Strip + no‐till	None + no Synthetic N	None	Sorghum–triticale double crop
NP, New York	CNV	Full width	Liquid	Broadcast incorporated, spring	Corn silage
	SHM‐1	Strip till	Liquid	Broadcast incorporated, spring	Corn silage–rye cover
	SHM‐2	Strip till	Evaporative solids	Broadcast solids, spring	Corn silage–rye cover
	SHM‐3	Strip till	Flocculated solids	Broadcast solids, spring	Corn silage–rye cover
	SHM‐4	Strip till	None	None	Corn silage–rye cover
TA, Texas	CNV‐1	Full width	Dried solids	Broadcast incorporated, spring	Sorghum silage
	CNV‐2	Full width	Slurry	Injected, spring	Sorghum silage
	CNV‐3	Full width	Slurry	Broadcast incorporated, spring	Sorghum silage
	CNV‐4	Full width	Synthetic N fertilizer	Broadcast incorporated, spring	Sorghum silage
	CNV no N control	Full width	None + no Synthetic N	None	Sorghum silage
	SHM‐1	Strip till	Dried solids	Broadcast incorporated, spring	Sorghum silage–wheat cover
	SHM‐2	Strip till	Slurry	Injected, spring	Sorghum silage–wheat cover
	SHM‐3	Strip till	Slurry	Broadcast incorporated, spring	Sorghum silage–wheat cover
	SHM‐4	Strip till	Synthetic	Broadcast incorporated, spring	Sorghum silage–wheat cover
	SHM no N control	Strip till	None + no Synthetic N	None	Sorghum silage–wheat cover
WA, Wisconsin	CNV	Full width	Liquid	Broadcast incorporated, after fall harvest	Corn silage
	SHM‐1	Strip till	Liquid	Injected, after fall harvest	Corn silage–rye cover
	SHM‐2	Strip till	Evaporative solids	Broadcast solids, spring and after fall harvest split applications	Corn silage–rye cover
	SHM‐3	Strip till	Flocculated solids	Broadcast solids, spring and after fall harvest split applications	Corn silage–rye cover
	SHM no N control	Strip till	None + no Synthetic N	None	Corn silage–rye cover
VA^a^, Vermont	CNV	Full width	Liquid	Broadcast incorporated, after fall harvest	Corn silage
	SHM	No‐till	Liquid	Injected, after fall harvest	Corn silage–rye cover

^a^VA also has a tile drainage treatment (with or without) within the conventional (CNV) and soil health management (SHM) plots.

**TABLE 5:** Measured manure properties from NO and NP sites in 2022. Values are means with standard errors. Liquid dairy manure is presented by site, since NO and NP occur on different dairy operations and use farm‐specific manure.

Material	Site	Solids (%)	Total N (%)	Total P (%)
Semi‐composted dairy manure	NO	27.9 ± 1.84	0.69 ± 0.10	0.10 ± 0.01
Flocculated solids	NP	87.7 ± 0.42	1.78 ± 0.05	1.06 ± 0.07
Evaporative solids	NP	85.1 ± 2.27	3.13 ± 0.09	0.98 ± 0.01

		Solids (%)	Total N (mg/L as received)	Total P (mg/L as received)
Liquid dairy manure	NO	4.09 ± 0.13	2406 ± 95	344 ± 10
Liquid dairy manure	NP	6.00 ± 2.73	2243 ± 51	277 ± 18

*Field‐scale* studies were designed to assess how adopting SHM systems on dairy farms changes soil health indicators, saturated hydraulic conductivity of the surface, soil carbon stocks, GHG emissions, and forage production at the landscape scale. They also assess how within‐field soil and hydrologic variability and connectivity affects SHM system effectiveness. This is being assessed through comparison of SHM fields with conventional (CNV) soil and manure management fields (Table 3). CNV soil and manure management is defined regionally, by common practices employed by commercial dairy management systems. The addition of SHM practices varies by location but generally includes using novel manure products, reducing tillage, and adding cover crops (Table 3). Tillage practices vary across regions, for example, in Vermont (site VB) the CNV field uses a chisel pass in the spring and fall and the SHM field uses a strip till pass in the spring, while in Wisconsin (site WP) the CNV field uses a disc at a 15‐cm depth and the SHM field is no‐till. Changes to measured outcomes are being monitored in SHM and CNV treatments over a 5‐year implementation period. Field‐scale studies provide an opportunity to show how novel SHM practices can be implemented at production‐scale.

Several studies have demonstrated that soil moisture is a major driver of soil health, GHG emissions, and agronomic outcomes and is influenced by hydrology and agricultural practices (Deines et al., 2019; Fan et al., 2017; Kaur et al., 2023; Ci et al., 2025). Additionally, both agronomic practices and changes in soil properties are hypothesized to influence soil moisture and hydrology, but few studies have demonstrated such effects (CAST, 2024). The field‐scale studies provide a unique opportunity to explicitly examine the interconnections between hydrology, management practices, and changing soil properties. For example, a shallow water table may overwhelm any local effects of SHM on forage yield or soil health indicators because the saturated conditions determine the soil biological activity and represent an overriding influence on crop productivity (Deines et al., 2024; Zipper et al., 2015). However, SHM can improve aggregate stability and reduce surface sealing, which would allow increased infiltration and decreases runoff on the landscape. If saturation in one part of the field was a consequence of a lack of infiltration in another part of the field, the effects of SHM on hydrology would only be measurable at the field‐scale that encompasses within‐field hydrologic gradients. Based on these considerations, we propose these overarching hypotheses:

1. Implementation of SHM systems will increase soil health because of increased soil carbon and improved soil structure. The changes to the soil hydraulic function will increase plant available water and alter the routing of water in a field (less runoff and surface redistribution).
2. Soil conditions at the field‐scale will be closer to optimal for plant growth when water storage and infiltration are increased in conjunction with reductions in runoff resulting in less variation in forage yield across the SHM fields.
3. Field‐scale changes to surface and subsurface hydrology will result in fewer poorly drained areas in a field leading to lower cumulative GHG emissions in the SHM fields.

**FIGURE 4:**
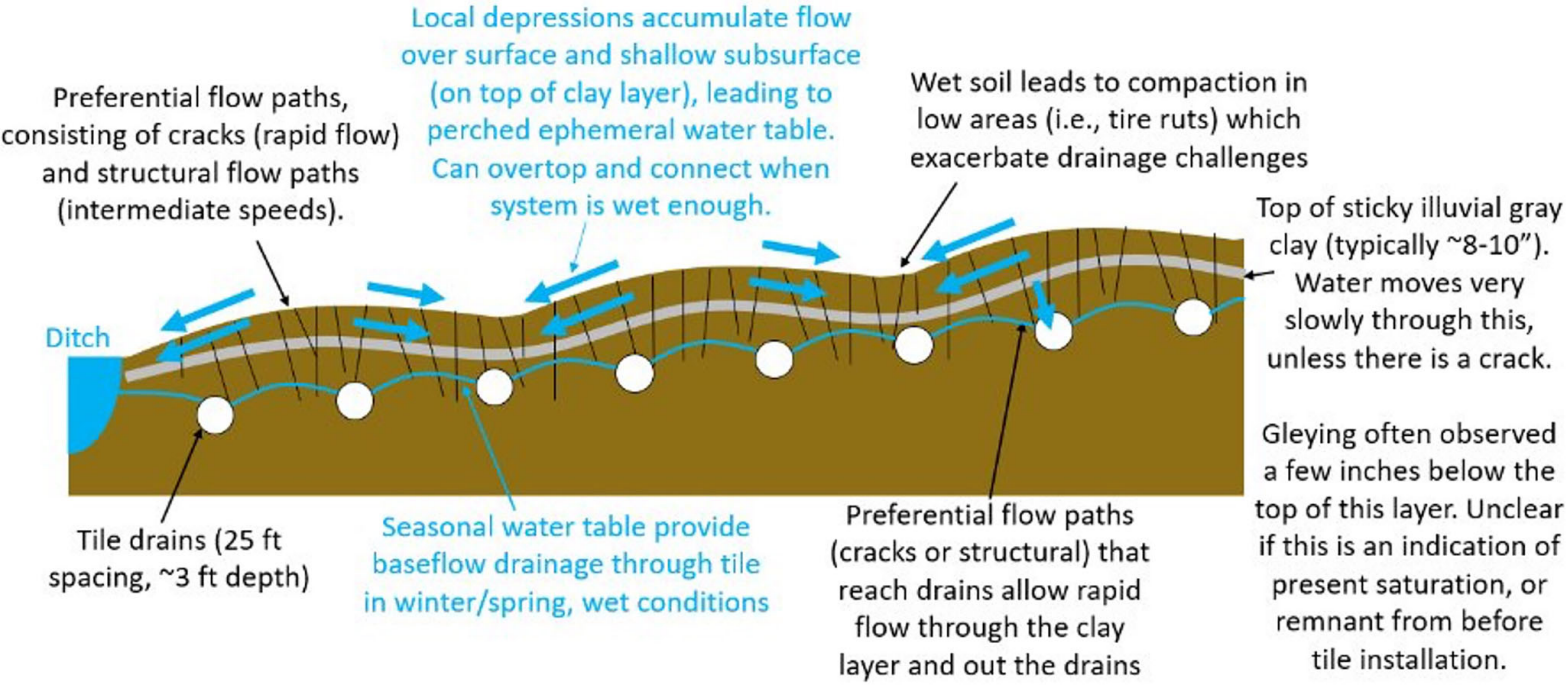
Conceptual models developed and used to select sampling sites at the field‐scale experiment in VB. The diagram presents soil hydrological considerations at the selected field.

Additionally, we expect that the impacts of SHM practices on soil and agronomic properties will vary across region, tillage, cropping, and novel manure treatments. Given the specific treatments used at each DSWR sites, there are additional hypotheses that can be tested based on the data collected from our experimental designs. To support hypothesis development, we have created conceptual models to describe these SHM‐hydrology interactions at individual sites. For example, at the field scale experiment in Vermont small differences in landscape position and preferential flow paths are the key drivers of the SHM‐hydrology relationship (Figure 4). Ultimately, changes in soil moisture, water flow, and water storage because of SHM need to be evaluated at the field scale due to within‐field hydrological connectivity. Sampling locations in the field‐scale studies were selected to span a range of hydrologic conditions within the field, for example, through local topographical high and low areas, or based on indicators of within‐field variability such as yield stability, which has been linked to variability in groundwater conditions in some regions (Deines et al., 2024; Zipper et al., 2015). In New York, yield stability zone maps (Cho et al., 2021) were used to identify sampling points so that the impact of enhanced soil management per management zone could be researched.

*Large‐plot* studies are randomized block experiments designed to assess combinations of soil health practices including no‐till or strip tillage, cover crops, and novel manure products on soil health, soil carbon stocks, GHG emissions, surface hydraulic conductivity, and forage production. Treatment combinations were selected based on regional context (Figure 2; e.g., California does not have a cover crop treatment because the commercial dairy farmer has a double‐crop forage operation). Large‐plot studies in New York (site NO) and Wisconsin (site WA) use a randomized complete block design, while the California study has a randomized block design augmented with treatments in replicated plots due to logistical constraints. The Texas study (site TA) uses a split‐plot design. Changes to soil health, soil carbon stocks, GHG emissions, and agronomic performance are monitored over a 5‐year implementation period. Table 4 details the CNV and SHM practices evaluated and management practices such as tillage practices vary across regions. For example, in Wisconsin (site WA), the CNV tillage is a cultivator in the spring and two chisel passes in the fall and the SHM tillage is a strip till pass in the spring and fall, whereas in Texas (site TA), the CNV tillage is a sweep plow and strip till system and the SHM tillage is strictly a strip till system. In California (site CP), the CNV tillage is one or two chisel passes in the fall followed by disking and mulching, which also happens in the spring. The SHM practices in CA utilized no‐till drill planting for triticale and sorghum except twice when sorghum was planted with a row planter after a pass with a strip tiller. The large‐plot studies were designed to test the following overarching hypotheses:

1. The combination of reduced tillage, cover cropping, and novel manure products will increase the values of soil health indicators (aggregate stability, soil carbon, potential carbon mineralization, and soil water holding capacity).
2. Increased nutrient use efficiency from tighter N cycling will occur in treatments with reduced tillage, cover cropping, and novel manure fertilizers.
3. At the end of the 5‐year monitoring period, agronomic outcomes (e.g., forage yield and forage quality) will be similar or better in SHM systems compared to CNV.

Similar to the field‐scale studies, we expect that the impacts of SHM practices on soil and agronomic properties will vary across tillage, cropping, and novel manure treatments and that treatment outcomes may differ across sites. Site‐specific hypotheses are being developed and tested by the research teams based in each region.

**FIGURE 5:**
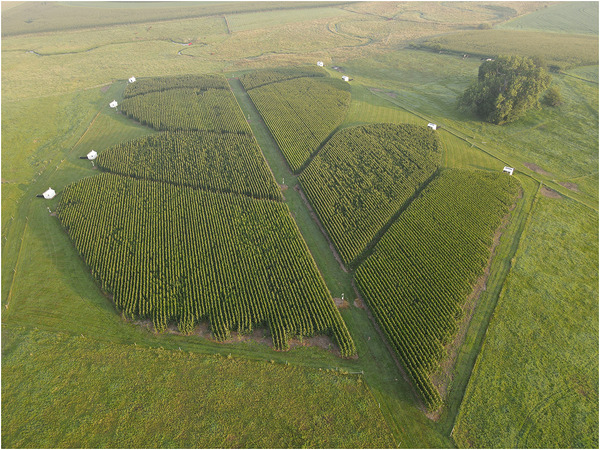
Aerial view of the fields at the Wisconsin Platteville (WI‐P) field‐scale experiment. There are eight catchments along the ridge, with two soil health management and two conventional catchments on each side of the ridge. Each field has a shed at the bottom of the catchment to collect runoff information and water samples (white sheds).

*Water quality and quantity* are undergoing additional focused monitoring at four sites: the Texas large‐plot site (TA; water quantity), the Wisconsin‐Platteville site (WP; water quality and quantity), and the Vermont large‐plot and field‐scale sites (VA, VB; water quality and quantity). At the Texas large‐plot site, access tubes were installed to use neutron probes to measure water use from a depth of 0–2.3 m at increments of 0.2 m starting at 0.1 m. Water use efficiency is calculated based on a per unit of water consumption including soil water from neutron probe measurements, irrigation, and rainfall. At the Wisconsin‐Platteville and Vermont sites, water quality and quantity are being assessed by measuring the fluxes of surface runoff and vadose zone leaching as well as concentrations of nitrogen, phosphorus, and sediment (see Table 6 measurement information). Vermont sites also include measurements from subsurface tile drainage water. Surface and subsurface (tile drain) discharge is being monitored using edge‐of‐field stations. Shallow groundwater is also being monitored using undisturbed soil core lysimeters at Wisconsin‐Platteville and piezometers at Vermont (Bergström, 1990; Persson & Bergström, 1991). Figure 5 shows the field treatment layout for Wisconsin‐Platteville and the runoff collection stations toward the bottom of the catchments.

**TABLE 6:** Measured water quantity and quality parameters for the Dairy Soil and Water Regeneration project along with the analytical method where appropriate for the large‐plot and field‐scale studies in Vermont and Wisconsin‐Platteville.

Sample type	Site(s) and location	Timing	Measurement	Method(s)	Reference
Water quantity	VA, VB, WP—Surface (edge of field); VA, VB—Subsurface (tile outlet)	Event‐based; biweekly and event‐based	Discharge rate	Stage‐discharge (H‐flume)	(Twombly et al., 2021)
				Stage‐discharge (compound weir); electromagnetic flow meter	(USDI‐BR, 1997)
Water quality	VA, VB, WP—Surface (edge of field); VA, VB—Subsurface (tile outlet); WP—Subsurface (lysimeter)	Event‐based; biweekly and event‐based; monthly	Total nitrogen	SM 4500‐N C modified, Lachat method 10‐107‐04‐1‐C	(APHA, 2017)
			Ammonium	10‐107‐06‐2‐a, sodium salicylate‐based method (EPA equivalent 350.1)	(APHA, 2017)
			Nitrate	SM 4500‐NO3 I, Lachat method 10‐107‐04‐1‐C	(APHA, 2017)
			Total phosphorus	SM 4500‐P manual digestion and flow injection analysis, Lachat method 10‐115‐01‐1‐F	(APHA, 2017)
			Soluble reactive phosphorus	Flow injection analysis, Lachat method 10‐115‐01‐1‐F	(APHA, 2017)
			Total suspended solids	SM 2540	(APHA, 2017)

*Off Dairy* studies are randomized complete block experiments designed to test how the application of manure to soils without a history of manure affects environmental and agronomic outcomes. A key benefit tied to the adoption of novel manure products is the generation of a source of nutrients that is more storable and transportable, allowing for the placement of manure more precisely based on where and when nutrients are needed. The off‐dairy project in California is on a commercial almond orchard. This design includes a no N control, synthetic N fertilizer, dairy manure compost, and a treatment with both synthetic N fertilizer and dairy manure compost. Plot sizes are 0.10 ha and span 30 trees. The off‐dairy project in ID, located at the USDA‐ARS in Kimberly, ID, includes a no manure control, a flocculated manure solid, and an evaporative manure solid that are applied to a field with or without a recent history of manure application. This site has a corn‐triticale double crop silage rotation and plots are 0.02 ha. Changes to soil health, soil carbon stocks, GHG emissions, surface hydraulic conductivity, and agronomics are being monitored over a 5‐year implementation period.

*Nitrogen replacement* studies are randomized complete block experiments designed to quantify the amount of commercial N replaced by manure products and evaluate impact of various manure products on forage yield. For these studies, there is a basal one‐time application of manure fertilizers at the beginning of the experiment. A series of commercial N fertilizer rates is superimposed on each basal manure treatment. These split‐plot experiments included the main treatment (manure type) applied to a block and commercial fertilizer rates randomized within each block. Liquid dairy manure, a flocculated manure solid, and an evaporative solid manure fertilizer were compared. Changes to forage quality and yield and soil N pools are being monitored over 2 years. These studies are being conducted in Idaho, New York, and Wisconsin (Figure 3). Average plot size for these experiments is 0.04 ha.

### 3.3 Modeling and communication

Task 3 was designed to (1) leverage the data collected from the studies to evaluate commonly used biophysical ecosystem‐service and soil carbon models and share results with model developers, and (2) share the learnings from the studies with stakeholder groups to support the adoption of SHM systems and use of novel manure products.

To evaluate the performance of a widely used biophysical model, COMET‐Farm (Li et al., 1992; https://comet‐farm.com), in estimating soil organic carbon in dairy feed production systems, modeled soil organic carbon estimates are compared with measured values from Task 1 and Task 2. COMET‐Farm was selected because (1) soil organic carbon is a primary model outcome, (2) it aligns with GHG inventory methods, and (3) it explicitly incorporates management effects. Specific objectives for the modeling component of this project include the following:

1. Assess model accuracy: Compare COMET‐Farm model estimates with observed values for soil carbon and available water holding capacity for the commercial farms sampled as part of Task 1.
2. Evaluate model performance over time: Compare COMET‐Farm estimates with observed values for soil carbon, available water holding capacity, and GHG emissions from Task 2's large‐plot and field‐scale experiments to determine how well the model captures changes in soil properties following the adoption of new practices.
3. Engage expert feedback: Consult agronomic modeling experts to interpret discrepancies between model estimates and observations and identify opportunities to improve model accuracy.
4. Support model refinement: Prepare a dataset that can be used by model authors and experts to facilitate calibration and enhancement of biophysical models.

Stakeholder outreach plans are ongoing and tailored to meet the needs of diverse audiences, including dairy farmers, cooperatives, agricultural consultants, funding partners, dairy organizations, and thought leaders in ecosystem markets and sustainability. Communication strategies are designed to foster interest in advanced SHM practices and novel manure products, while also supporting collaborating farmers in sharing their experiences. Outreach mechanisms include a publicly accessible project website, webinars, conference presentations, panel discussions, annual progress summaries, media engagement, videos highlighting farmer and researcher perspectives, and field days.

This paper serves as a brief introduction to DSWR. For project updates visit https://dairysoilwater.org.

## AUTHOR CONTRIBUTIONS

**Mara L. Cloutier**: Conceptualization; methodology; project administration; visualization; writing—original draft; writing—review and editing. **Sam Zipper**: Conceptualization; formal analysis; methodology; visualization; writing—original draft; writing—review and editing. **Daniel Liptzin**: Conceptualization; methodology; visualization; writing—original draft; writing—review and editing. **E. Carol Adair**: Methodology; writing—review and editing. **Brent W. Auvermann**: Funding acquisition; visualization; writing—review and editing. **Jourdan M. Bell**: Methodology; writing—review and editing. **Bert Bock**: Conceptualization; funding acquisition; methodology; visualization; writing—original draft; writing—review and editing. **Carolina B. Brandani**: Methodology; writing—review and editing. **Dennis Busch**: Funding acquisition; methodology; writing—review and editing. **Nicholas E. Clark**: Methodology; writing—review and editing. **Karl J. Czymmek**: Methodology; writing—review and editing. **Joshua W. Faulkner**: Funding acquisition; methodology; visualization; writing—review and editing. **Adam C. von Haden**: Methodology; visualization; writing—review and editing. **William R. Horwath**: Methodology; writing—review and editing. **Randall D. Jackson**: Funding acquisition; methodology; visualization; writing—review and editing. **Quirine M. Ketterings**: Funding acquisition; methodology; visualization; writing—review and editing. **Sat Darshan S. Khalsa**: Funding acquisition; writing—review and editing. **Deanne Meyer**: Funding acquisition; methodology; writing—review and editing. **Gregg R. Sanford**: Conceptualization; methodology; visualization; writing—review and editing. **James Wallace**: Conceptualization; funding acquisition; project administration; writing—review and editing. **Reza K. Afshar**: Project administration; visualization; writing—review and editing. **Cristine L. S. Morgan**: Funding acquisition; methodology; project administration; writing—review and editing.

## CONFLICT OF INTEREST STATEMENT

The authors declare no conflicts of interest.
